# Eco-Conscious Orthodontics: A Greener Approach to Dental Care

**DOI:** 10.7759/cureus.78809

**Published:** 2025-02-10

**Authors:** Priya Shrivastava, Chanamallappa Ganiger, Renuka Pawar, Sandesh Phaphe, Yusuf Ronad, Pratap Mane

**Affiliations:** 1 Department of Orthodontics and Dentofacial Orthopedics, School of Dental Sciences, Krishna Institute of Medical Sciences, Karad, IND

**Keywords:** advice, ask, assess, eco-friendly dentistry, green dentistry, green orthodontics, recycle, reduce, rethink, reuse

## Abstract

With the advancing world, where the medical and dental fields are progressing every day toward better and finer diagnosis and treatment modalities to enhance and provide better standards of life, it becomes important for medical and dental professionals to consider the environment and apply environmental sustainability measures moving towards a better future. One such practice is green dentistry, where “reduce, reuse, rethink, and recycle” are being implemented and, similarly, “eco-friendly” dentistry, which recommends the use of “ask, assess, advice, and assist.” With the same concept of keeping in the limelight, green orthodontics gives us an idea of reducing, reusing, rethinking, and recycling materials and products to bring about a more sustainable and better future.

## Introduction and background

The term "eco-friendly dentistry" was first used and patented by Dr. Goran Kralj, Dr. Steven Koos, and Mladen Kralj, the founders of ORA Dental Studio, a green group of dental practitioners [1]. With the advent of concepts dependent on the principles of sustainable development, which can be defined as the development that "meets the needs of the present without compromising the ability of future generations to meet their own needs," the dental field has, step by step, developed in terms of diagnosis and treatment planning, materials, techniques, and procedures [1].

However, this concept carries a huge responsibility for dental and medical practitioners to protect natural resources and reduce the effects of lethal waste generated from these practices. Dental practitioners can help the principles of green dentistry by making a few changes in their practice, leading to a sustainable future. Newer techniques, products, and technologies for cleaner, greener dental practices are emerging, allowing practitioners to mediate/eliminate, much of this waste. However, there are a lot of things to be done [1].

Although each dentist produces only a small portion of unfriendly waste, the entire profession's total waste significantly impacts the environment. "Green dentistry" commonly known as eco-friendly dentistry, has been described by Dr. Ali Farhani as an "approach to dentistry that implements sustainable practices by keeping resource consumption in line with nature’s economy, by safeguarding the external environment by eliminating or reducing outgoing wastes and by promoting the well-being of all those in the clinical environment by conscious reduction of the chemicals in the breathable air" [1].

**Figure 1:**
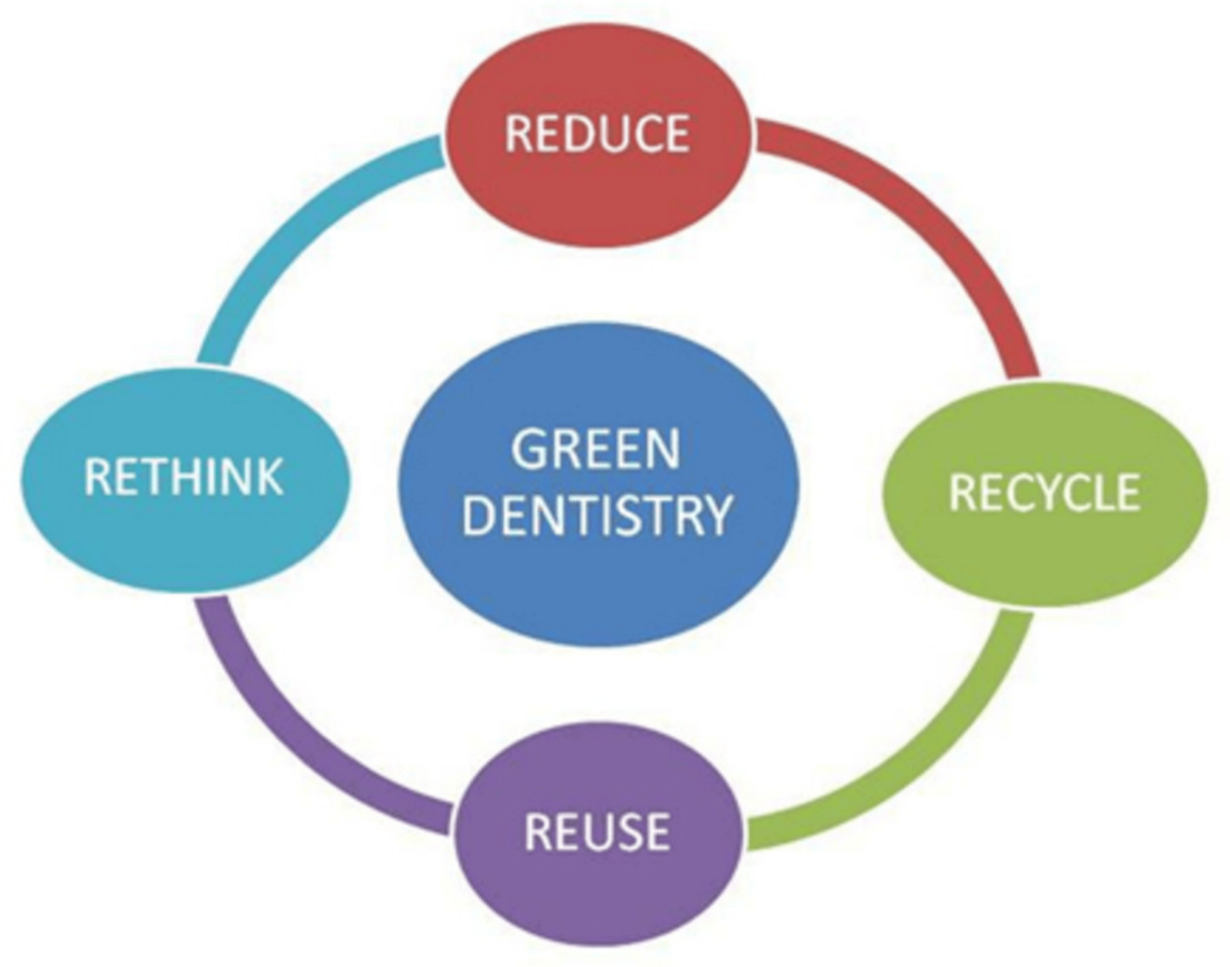
The 4 Rs of green dentistry Source: Ref. [2]

Green dentistry mainly focuses on the four "Rs," that is, rethink, reduce, reuse, and recycle (Figure 1) [2].

They also describe that "eco-friendly dentistry" may safeguard the immediate health of patients and team members, the community at large, and the world's health by using green design and operations, community, and the environment.

The eco-friendly model for dentistry includes four "As": ask, assess, advise, and assist the dentists.

Ask: Collect all the details from the dentist on his routine dental practices;

Assess: Assess what all practices can be modified/altered towards promoting eco‑friendly dentistry;

Advice: A clear set of guidelines that can be followed; and

Assist: Assist in preparing a framework/routine as per the environmental conditions at the place of the dental clinic/hospital [3].

The abovementioned terms "eco-friendly dentistry and green dentistry" motivate each specialization of dentistry to look towards the environment, one such specialization is "orthodontics and dentofacial orthopedics," which tends to focus towards "green orthodontics."

Thus, green orthodontics is considered that the green color is thought to be the most peaceful and quiet hue and to have therapeutic qualities. Green is also linked to renewal, development, optimism, steadiness, fortitude, better vision, and hope [4]. Applying the same idea to reuse, rethink, rejuvenate, and reduce the risk of environmental hazards through safer orthodontic practices might be considered as practicing orthodontics in a greener way, that is, "green orthodontics."

Before practicing green orthodontics, it becomes important to focus on what are the causes that inhibit operators from practicing green orthodontics and what can be done to reduce/prevent generating such wastes. There are a few such examples such as alginate impressions and gypsum for preparing working and model casts. These gypsum products when treated with a 20% concentration of ammonium bicarbonate solution can help with the quick and environmentally beneficial disintegration of such gypsum waste, which later can be used as fire extinguisher powder, nitrogen fertilizer, and in wood pulp, textile, and pharmaceutical industries [4].

**Figure 2:**
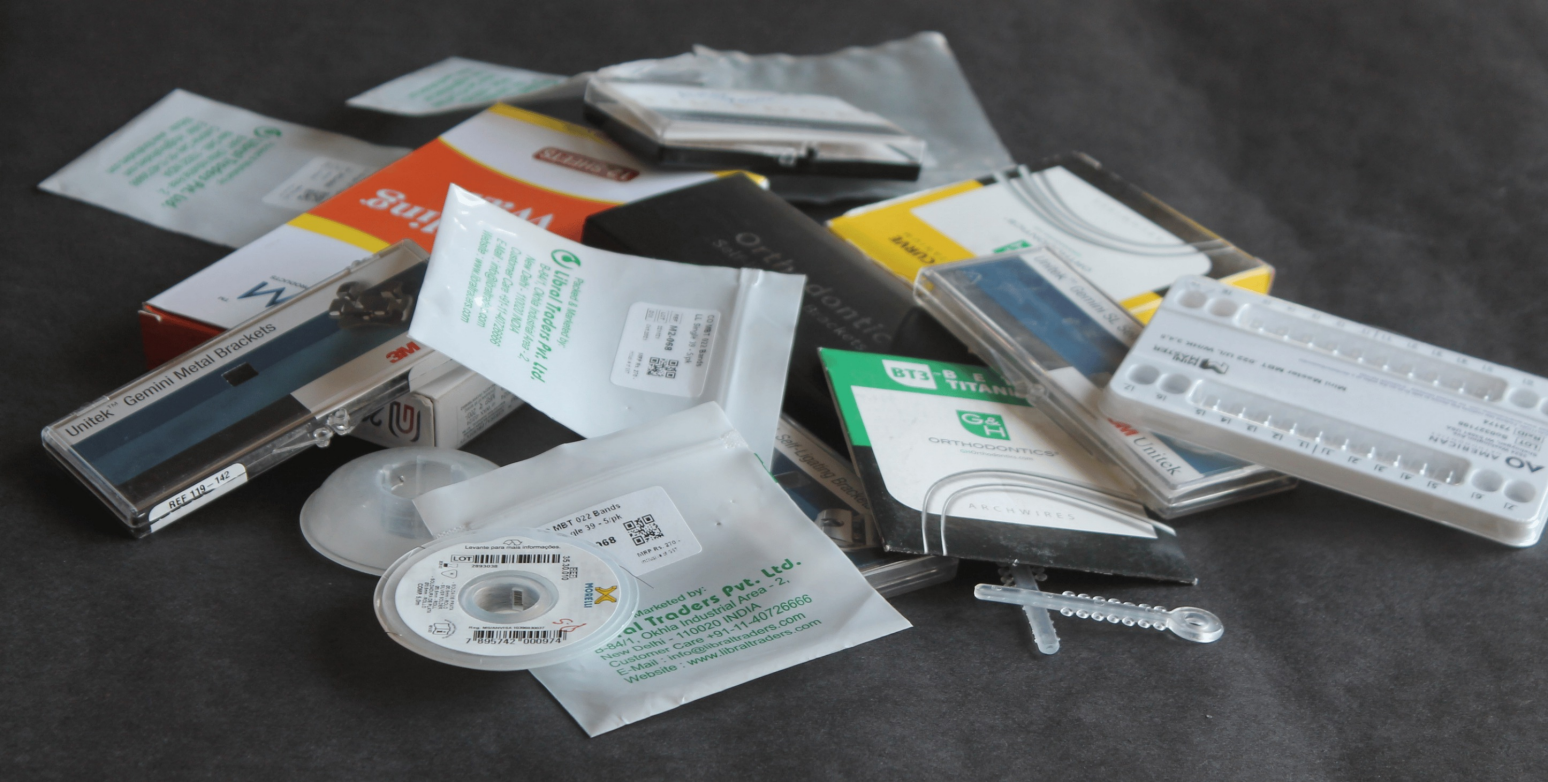
Orthodontic material packaging Source: Ref. [4]

One other example of orthodontic waste is orthodontic material packaging, which becomes garbage [4] (Figure 2). To reduce this garbage, biodegradable material packaging should be done.

Another most common waste within dental clinics is paper. Dentists could be more environmentally friendly by switching from a paper-based records system to a computer-based records system [4], which can be used for saving patient-related data and for diagnosing and treatment planning and manufacturing of appliances too.

An alternative solution that can be used by orthodontists is CAD/CAM (computer-aided design/computer-aided manufacturing), intra-oral scanners, and 3D printing that helps in diagnosing, treating, and manufacturing precise appliances, which can be beneficial, less time-consuming, and more patient-friendly and significantly reducing the waste.

## Review

For sustainable orthodontic practices, various accessories used in orthodontics such as elastomeric chains (e-chain), temporary anchorage devices (TADS), orthodontic brackets, etc. are being used, and various studies on their reuse and recycling have been done, leading us towards less material waste and more sustainable practices.

In their article on sustainable orthodontics, Pithon et al. concluded that the team's individual and collective awareness and efforts make it possible to implement sustainable orthodontics, reducing costs and safeguarding the environment. By minimizing the environmental impact of orthodontic practices, the team contributes to the planet's recovery, including taking precautions when utilizing natural resources for treatment​​​​ [5].

He mentioned fewer sustainable practices and their benefit, which will help orthodontists practice in a more sustainable way, as depicted in Table 1 [5].

**Table 1:** Alternative and sustainable orthodontic practices and its benefit Source: Ref. [5]

What is done	Sustainable practice	Benefit
Orthodontic accessories sold in conventional packaging	Brackets sold in receptacles with a larger quantity of accessories, with these receptacles being manufactured of a recyclable product	Elimination of packaging made of plastic materials, being replaced with biodegradable materials
Adhesive systems with acid etching	Self-etching adhesive systems	Lower water consumption due to no need for washing and drying, with same clinical efficacy
Conventional brackets	Self-ligating brackets	Less chair time and eliminates the use of elastomers
Use of non-sterilizable orthodontic archwires	Use of orthodontic archwires capable of being sterilized	Reduce discard of solid residues that may have been contaminated before use in the patient
Re-bond new brackets when they de-bond during treatment	Recycle brackets by roughening their base with aluminum oxide and performing new bonding	Eliminate solid residues that would go to the trash can, making it possible for them to have a longer useful life
Light polymerization with conventional halogen or LED appliances	Ultra-rapid LED light	Shorter chair time and use of LED lamp with low energy consumption
Use of synthetic intermaxillary elastics	Use of elastics made of latex	Latex is extracted from a tree; consequently, there is a need to cultivate trees; therefore, the more widespread the use of latex, the larger the number of trees
The use of a new mini-implant in a patient who needs to replace the one in use	Sterilization and use of the same mini-implant that was removed in the same patient	Reduction of solid residues that are constituents of the mini-implant

Notaroberto et al. mentioned orthodontic elastics, which have been commonly used as orthodontic rings for correction of space closure and employed for tooth separation in a variety of ways prior to the establishment of inter-arch relationships [6].

Natural latex-based materials and materials free of latex or synthetics are the two types of fabrication materials. A vegetable extract is used to make rubber/latex elastics, which are then manufactured till the end product is obtained. The proportion of patients with latex allergy and occupationally exposed groups is linked to the indication for the use of synthetic elastics.

The literature has a lot of information on laboratory tests examining the mechanical characteristics of elastics with and without latex. These findings demonstrate that latex-containing elastics degrade less over time than latex-free ones. In addition, this research has shown that the first day of use is when elastics have the largest force degradation. However, the actual oral conditions are extremely intricate, and various studies that are performed in vitro may not be able to produce precise and accurate results.

There are multiple elements present in the oral cavity that play a key role in maintaining the oral environment, such as variations in pH, temperature, enzymes, and microbes, and the patient’s oral hygiene routine. All of these factors, including intermaxillary elastic elongation in these media, result in the decay of force in the orthodontic elastics. Therefore, it is necessary to conduct in situ/in vivo studies in order to contemplate and fully understand the effects of elastics within the oral medium with different existing factors [6].

Pithon et al. did a clinical study on the loss of force of latex and non-latex intermaxillary elastics and came up with some interesting facts. They found that latex and non-latex elastics (1/8 diameter) exhibited highly variable and statistically significant differences at all evaluation times. In contrast, 1/4 and 5/16 elastics, whether latex or non-latex, showed very minimal changes in force delivery, even after 24 hours of use [7].

Coming on to orthodontic brackets, various works related to the recycling of brackets have been done, and keeping in view the debonding procedure, the potential exists for distortion/damage to both metal and ceramic brackets and their mesh bases, which provides retention on tooth surfaces. Therefore, whether a commercial recycler is used or the orthodontic practitioner recycles it, in-house proper care should be taken to maintain and not alter the bracket and base combination. Once each bracket and base combination has been achieved and approved, the recycling method used should remain the same and not change the physical properties of this appliance. In addition, bracket specifications such as tip, torque values, and physical properties must be verified with those stated by the manufacturer.

Buchman did a study on the recycling of metallic direct-bond orthodontic brackets and concluded that recycling/reusing of metallic orthodontic appliances can be advantageous and of great significance, both economically and ecologically provided that orthodontists are knowledgeable and want to make conscious efforts in this expanding field [8].

Dr. Machen, an orthodontist and attorney in Pittsburgh, PA, contributed to research on "orthodontic bracket recycling," with his comments published in a journal [9]. He noted that the mechanical properties of metallic brackets remained unchanged when the orthodontic adhesive was removed from the brackets using solvent stripping at temperatures below 100°C, followed by a heat treatment at 250°C for sterilization [9].

He also mentioned that these brackets have been used in the intra-oral environment for months or even years and that special care must be taken during their sterilization before reuse. Since the solvent formation in one process is unknown, relying solely on the solvent and heat treatment below 100°C may not effectively sterilize the brackets. Therefore, a separate sterilization cycle is necessary, followed by proper packaging to prevent contamination, ensuring that patients receiving these recycled brackets are protected from any harmful contaminants that could affect their health [9].

Bahnasi et al. did a study on the recycling of stainless steel (SS) orthodontic brackets and compared the shear bond strength (SBS) of newer brackets with that of using brackets, which were recycled using 50-μm aluminum oxide, grinding, thermal, or chemical methods [10]. SBS can be defined as the maximum force that an orthodontic adhesive joint can tolerate and resist before fracture. Their findings are as follows: (1) bracket recycling using 50-μm aluminum oxide (Al2O3) powder did not alter the SBS of stainless steel brackets and certainly can be used as another option than new brackets, which will be cost-effective; (2) bracket recycling procedures using grinding, thermal or chemical methods depicted reduced SBS as recommended by Reynolds; and (3) in the case of using repeated recycled brackets by sandblasting, enhanced bond strength was observed with bonding agent application to the brackets.

Gupta et al. did an in vitro study on the SBS of SS brackets with three innovative recycling methods, namely, sandblasting, direct flaming, and acid bath solution, and compared it with that of newer brackets [11]. Their findings are as follows: (1) the SBS of newer brackets is significantly increased than the recycled brackets; (2) brackets that were recycled using sandblasting with 90-µm aluminum oxide (Al2O3) particle air-abrasion depicted significantly increased SBS in comparison to direct flaming/sandblasting and direct flaming/sandblasting/acid bath solution; and (3) sandblasting with 90-µm aluminum oxide particle air abrasion is the simplest, most efficient, and, hence, the preferred method of recycling debonded brackets.

Yassaei et al. did a study on ceramic bracket recycling and its effects using Er-YAG laser and sandblasting [12]. Their findings are as follows: (1) both Er:YAG laser and sandblasting were effective in reconditioning and providing mechanically retentive ceramic brackets, which could effectively adhere to the tooth surface without fracture; (2) the shear bond strength of brackets recycled with Er:YAG laser and new brackets did not show much difference; (3) Er:YAG laser recycling could effectively and efficiently remove the adhesives with not much damage to the base of the bracket, unlike sandblasting. Although sandblasted brackets showed maximum strength, but brackets bases were altered; and (4) Er:YAG laser recycling showed the brackets with an appropriate shear bond strength that is of clinical significance. Most of the recycled specimens showed an adhesive remnant index (ARI) score, which was 3. ARI is a scoring system used to assess the amount of adhesive left on a tooth after debonding

Mattos et al. did a study on the effects of autoclaved mini-implants for fracture torque resistance, which are used in orthodontics for anchorage [13]. Their findings are as follows: (1) not many relevant effects on the fracture torque resistance of these autoclaved mini-implants were observed, and (2) mini-implants from different manufacturers may show remarkably significant differences in their properties of fracture resistance. The fracture torque resistance of mini-implants, also known as the torque at which a mini implant breaks, varies depending on the implant design and diameter, but generally, larger diameter mini-implants have significantly higher fracture torque resistance, meaning that they are more resistant to breaking when subjected to force.

Yadav et al. did a study on the mechanical properties of nickel-titanium (NITI) wires after recycling and their reuse after cold sterilization and concluded that there was a decrease in stiffness shown by the recycled NITI wires after six weeks of clinical use. The findings of his study supported the recycle/reuse of NITI wires even after six weeks of use in oral conditions, followed by cold sterilization by putting wires in 2% acidic glutaraldehyde for a duration of 10 hours, and did not show any topographical changes of pitting on surfaces of NITI wires [14].

Macrì et al. did a study on clear aligner therapy and its implications on the environment and came up with some alarming and insightful conclusions [15]. Their findings are as follows: (1) More studies should be done to thoroughly understand the consequences of nano- and microplastics (NMPs) on the mankind and its effects. More researches should be done to quantify the amount of NMPs that may be produced from the fabrication and use of these aligners, and recognizing the environmental impact of the failed prints of NMPs and post-processing waste, which can turn out to be pivotal. (2) Understanding the advantages of fixed therapy as a viable alternative treatment option, rather than going for clear aligner therapy, which might prolong the treatment time, is also important. (3) Educating and making patients aware of the proper usage and right way of disposal of aligners is essential to minimize waste. Implementation of strategies such as dividing deliveries into multiple phases/steps and reducing packaging can help lessen the number of unused aligners. (4) Incorporating recycled materials into 3D printing processes can boost the idea of eco-sustainable future. Finding new types of plastics that are beneficial for 3D printing aligners, such as biodegradable or environment friendly polymers, is commonsensical. (5) Detailed researching and identification of microbes which are capable of degrading/disintegrating plastic polymers can also help in developing sustainable disposal methods. (6) Campaigns promoting right way of disposal and recycling techniques for aligners should be there, with collection containers situated near flagship locations for easy recycling. (7) Companies can collect already used aligners for recycling and later use them as a medium of fuel for electricity or heat production. (8) By acquiring new ways of recycling of aligners, orthodontic practitioners can help move to a better and eco-conscious sustainable future and lessen the impact of orthodontic treatment waste on environment.

All these above studies give us a hint of reducing, reusing, rethinking, and recycling orthodontic auxiliaries and accessories, which in turn helps in reducing the material waste, cost-cutting, producing less material waste, and bringing environment-friendly solutions and providing the main focus on effective treatment results.

## Conclusions

The abovementioned studies give us a hope of managing orthodontic practices with less material waste and a more environment-friendly, eco-conscious, and sustainable future provided that there should be more precise and accurate in vivo research and innovative methods.
